# Clinical features and treatment of heterotopic pancreas in children: a multi-center retrospective study

**DOI:** 10.1007/s00383-024-05722-z

**Published:** 2024-05-29

**Authors:** Xiaofeng Yang, Chen Liu, Shuai Sun, Chao Dong, Shanshan Zhao, Zaitun M. Bokhary, Na Liu, Jinghua Wu, Guojian Ding, Shisong Zhang, Lei Geng, Hongzhen Liu, Tingliang Fu, Xiangqian Gao, Qiong Niu

**Affiliations:** 1 Department of Pediatric Surgery, Binzhou Medical University Hospital, Binzhou, China; 2 Department of Surgery, Shanghai Children’s Hospital, Shanghai Jiao Tong University, Shanghai, China; 3 Department of General Surgery, Zibo Hospital of Shandong Yiyang Health Industry Development Group Co, Ltd, Zibo, China; 4 Department of Pediatric Surgery, Muhimbili National Hospital, Dar Es Salaam, Tanzania; 5 Department of Pathology, Qilu Hospital of Shandong University Dezhou Hospital, Dezhou, China; 6 Department of Pediatric Surgery, Qilu Hospital of Shandong University Dezhou Hospital, Dezhou, China; 7 Department of Pediatric Surgery, Children’s Hospital Affiliated to Shandong University, Jinan, China; 8 Department of Pathology, Binzhou Medical University Hospital, Binzhou, China; 9 Department of Gastroenterology, Binzhou Medical University Hospital, Binzhou, China

**Keywords:** Heterotopic pancreas, Ectopic pancreas, Pancreatic rest, Meckel’s diverticulum, Treatment, Children

## Abstract

**Objective:**

Heterotopic pancreas, an uncommon condition in children, can present with diagnostic and treatment challenges. This study aimed to evaluate the clinical features and treatment options for this disorder in pediatric patients.

**Methods:**

We conducted a retrospective analysis, including patients diagnosed with heterotopic pancreas at four tertiary hospitals between January 2000 and June 2022. Patients were categorized into symptomatic and asymptomatic groups based on clinical presentation. Clinical parameters, including age at surgery, lesion size and site, surgical or endoscopic approach, pathological findings, and outcome, were statistically analyzed.

**Results:**

The study included 88 patients with heterotopic pancreas. Among them, 22 were symptomatic, and 41 were aged one year or younger. The heterotopic pancreas was commonly located in Meckel’s diverticulum (46.59%), jejunum (20.45%), umbilicus (10.23%),ileum (7.95%), and stomach (6.82%). Sixty-six patients had concomitant diseases. Thirty-three patients had heterotopic pancreas located in the Meckel’s diverticulum, with 80.49% of cases accompanied by gastric mucosa heterotopia (GMH). Patients without accompanying GMH had a higher prevalence of heterotopic pancreas-related symptoms (75%). Treatment modalities included removal of the lesions by open surgery, laparoscopic or laparoscopic assisted surgery, or endoscopic surgery based on patient’s age, the lesion site and size, and coexisting diseases.

**Conclusions:**

Only one-fourth of the patients with heterotopic pancreas presented with symptoms. Those located in the Meckel’s diverticulum have commonly accompanying GMH. Open surgical, laparoscopic surgical or endoscopic resection of the heterotopic pancreas is recommended due to potential complications. Future prospective multicenter studies are warranted to establish rational treatment options.

## Introduction

Pancreatic tissues located at unusual sites, exhibiting histological structures similar to those of normal pancreas, yet lacking anatomical relation to the normal pancreas, and direct blood vessel connections to the normal pancreas are termed “heterotopic pancreas, aberrant pancreas, or ectopic pancreas” [1, 2]. Its pathogenesis is attributed to abnormal developmental regulatory mechanisms [3]. Heterotopic pancreas can be found in various locations, including the stomach, duodenum, jejunum, Meckel’s diverticulum, duplication or other sites [4, 5]. While heterotopic pancreas often remains asymptomatic and is incidentally detected during gastroscopy or abdominal surgery for other unrelated causes [6], it can manifest with symptoms such as acute or chronic inflammation, bleeding, and intestinal obstruction [4, 5]. Moreover, reports exist of malignant transformation to adenocarcinoma or acinar cell carcinoma originating from heterotopic pancreas [7]. The diagnosis and optimal treatment of this condition pose challenges for clinicians in the pediatric population [8, 9]. The aim of this study was to explore the clinical features and treatment options for heterotopic pancreas in children.

## Materials and methods

Electronic medical records of pediatric patients with pathologically confirmed heterotopic pancreas were retrospectively collected from four tertiary hospitals between January 2000 and June 2022, using the search terms ‘‘ectopic pancreas, heterotopic pancreas, and/or adenomyoma”.

The inclusion criteria were as follows: (1) patients with confirmed pancreatic tissue in other sites without any connection with the normal pancreas (2) patients aged ≤ 18 years with symptom onset related to heterotopic pancreas or incidentally found due to other abdominal surgery or endoscopy screeningand (3) pathologically confirmed diagnosis. The exclusion criteria included patients aged > 18 years with symptom onset of heterotopic pancreas, and patients with a suspected diagnosis of heterotopic pancreas that was not confirmed pathologically.

Clinical data, including sex, age, clinical presentation, lesion size and site, coexisting diseases, treatment approach, Heinrich pathological type, and occurrence of intra- and postoperative complications were collected. Patients with symptoms strongly associated with heterotopic pancreas were considered symptomatic, whereas those whose lesions were incidentally found in other diseases were considered asymptomatic. Patients presenting with symptoms due to the mechanical causes of Meckel’s diverticulum or duplication cysts were classified as asymptomatic [5, 10].

Regarding pathological evaluation, sections were reviewed by senior pathologists from their hospitals and categorized into three types based on the Heinrich classification [1, 6, 11]. Briefly, type I consists of three components: pancreatic acini, ducts, and islet cellstype II is composed of acini and pancreatic ductsand type III contains only pancreatic ducts. Gastrointestinal adenomyomas, consisting of ducts and smooth muscle bundles, are categorized as type III [11, 12].

The patients were divided into symptomatic and asymptomatic groups based on their clinical presentation. Categorical variables are shown as numbers and percentages and were statistically analyzed using Fisher’s exact test. All statistical analyses were performed using SPSS version 22 software (IBM Corp., Armonk, NY, USA). A *p* < 0.05 was considered statistically significant. The present study was conducted in accordance with the Declaration of Helsinki and approved by the Ethics Committee of Binzhou Medical University Hospital (No. 20210120-01). Written informed consent for the use of their data for scientific purposes was obtained from the parents/legal guardians (s) of all children involved in the study.

## Results

Eighty-eight patients with pathologically confirmed heterotopic pancreas met the inclusion criteria. Patient characteristics are summarized in Table 1. The results indicate that 41 (46.59%) patients were aged 1 year or younger, with a male-to-female ratio of 1.84:1. Twenty-two patients (25%) exhibited symptoms related to a heterotopic pancreas. The lesions were predominantly found in the Meckel’s diverticulum (46.59%), jejunum (20.45%), ileum (7.96%), stomach (6.82%), and umbilicus (10.23%). Three-fourths of the patients had comorbidities such as gastrointestinal bleeding, intussusception, omphalomesenteric duct anomalies, jejunal or ileal atresia/stenosis, and acute abdomen (malrotation with gut volvulus, appendicitis, and intestinal perforations).

**Table 1:** The characteristics of heterotopic pancreas in pediatric patients

Variables	Number of patients	Percentage
Total patients	*n* = 88	100
Age at surgery		
≤ 1 yr	41	46.59
> 1–5 yr	32	36.36
> 5–18 yr	15	17.05
Male: female	57:31 (1.84:1)	
Symptomatic/asymptomatic	22/66	25.0
≤ 1 y	8/33	19.51
> 1–5 y	6/26	18.75
> 5–18 y	8/7	53.33
Locations		
Stomach	6	6.82
Duodenum	2	2.27
Jejunum	18	20.45
Ileum	7	7.96
Mesentery	2	2.27
Meckel’s diverticulum	41	46.59
Umbilical site	9	10.23
Biliary tract	2	2.27
Mediastinum	1	1.14
Concomitant disease (s)	66	73.86
Gastrointestinal bleeding	25	28.41
Intussusception	10	11.36
Omphalo-mesenteric duct	7	7.96
Malrotation	5	5.68
Congenital choledochal cyst	4	4.54
Appendicitis	3	3.41
Jejunal or ileal atresia/stenosis	3	3.41
Duplication cyst	2	2.27
Small intestinal perforation	2	2.27
Urachal cyst	2	2.27
Esophageal atresia	1	1.14
Biliary atresia	1	1.14
Neuroblastoma	1	1.14
Treatment approach		
Open surgery	76	86.36
Laparoscopy	8	9.09
Gastroscopy	4	4.55
Heinrich’s classification		
I	82*	93.18
II	3	3.41
III	2	2.27
Not available	1	1.14

*one case concomitant with adenomyoma

Eighty-two cases (93.18%) were classified as Heinrich type I, while three (3.41%) as type II, and two (2.27%) as type III. There was no evidence of neoplasia found in this case series. The clinical spectrum of heterotopic pancreas varies from asymptomatic to mild abdominal pain to severe peritonitis caused by gastrointestinal perforations. The occurrence of symptoms in patients aged ≤ 1 year, > 1–5 years, and > 5–18 years was 8/41 (19.51%), 6/32 (18.75%), and 8/15 (53.33%), respectively.

As depicted in Table 2, the heterotopic pancreas within Meckel’s diverticulum is typically located at the apex of the diverticular lumen, with a lesion size less than 1 cm in maximum diameter. Intestinal bleeding and intussusception were common symptoms. Heterotopic pancreas accompanying gastric mucosa heterotopia (GMH) (80.49%) was found in 33 patients, and those without GMH had a higher prevalence of heterotopic pancreas related symptoms (75%), *p* < 0.001.

**Table 2:** Clinical features of Meckel’s diverticulum concomitant with heterotopic pancreas

Variables	*n*	Symptomatic (*n*, %)	Asymptomatic (*n*, %)	*P* value
Total patients	41	6 (14.63)	35 (85.37)	
Age at surgery (year)				
≤ 1	19	3 (50)	16 (45.71)	0.531
> 1–5	16	3 (50)	13 (37.14)	
> 5–18	6	0	6(17.05)	
Male: female	32:9	3:3	29:6	0.207
Locations				
Apex of the diverticular lumen	34	4 (66.67)	30 (85.71)	0.391
Diverticular mesentery	1	0	1 (2.86)	
Diverticular wall	1	0	1 (2.86)	
Not available	5	2 (33.33)	3 (8.57)	
Size of the long diameter (mm)				
< 5	19	2 (33.33)	16 (45.71)	0.065
5–10	20	2 (33.33)	17 (48.57)	
> 10	1	2 (33.33)	0	
Not available	1	0	2 (5.71)	
Initial diagnosis				
Bleeding	25	3 (50)	22 (62.86)	0.077
Intussusception	8	0	8 (22.86)	
Internal hernia	3	1 (16.67)	2 (5.71)	
Perforation	1	1 (16.67)	0	
Others	4	1 (16.67)	3 (8.57)	
GMH	33	0	33 (100.0)	** < 0.001**
Without GMH	8	6 (75.0)	2 (25.0)	
Surgical approach				
Open surgery	37	5 (83.33)	32 (91.43)	> 0.99
Laparoscopic surgery	4	1 (16.67)	3 (8.57)	

Bold indicates statistical significance

*GMH* gastric mucosa heterotopia

Coexisting diseases, including Meckel’s diverticulum, gut malrotation, intestinal stenosis/atresia, intussusception, duplication cyst, gastrointestinal perforation, biliary atresia, congenital choledochal cyst, a peri-umbilical mass, mediastinal mass, and neuroblastoma, were diagnosed by radiography, upper gastrointestinal series (UGIs), ultrasonography, or endoscopic ultrasonography.

**Fig. 1:**
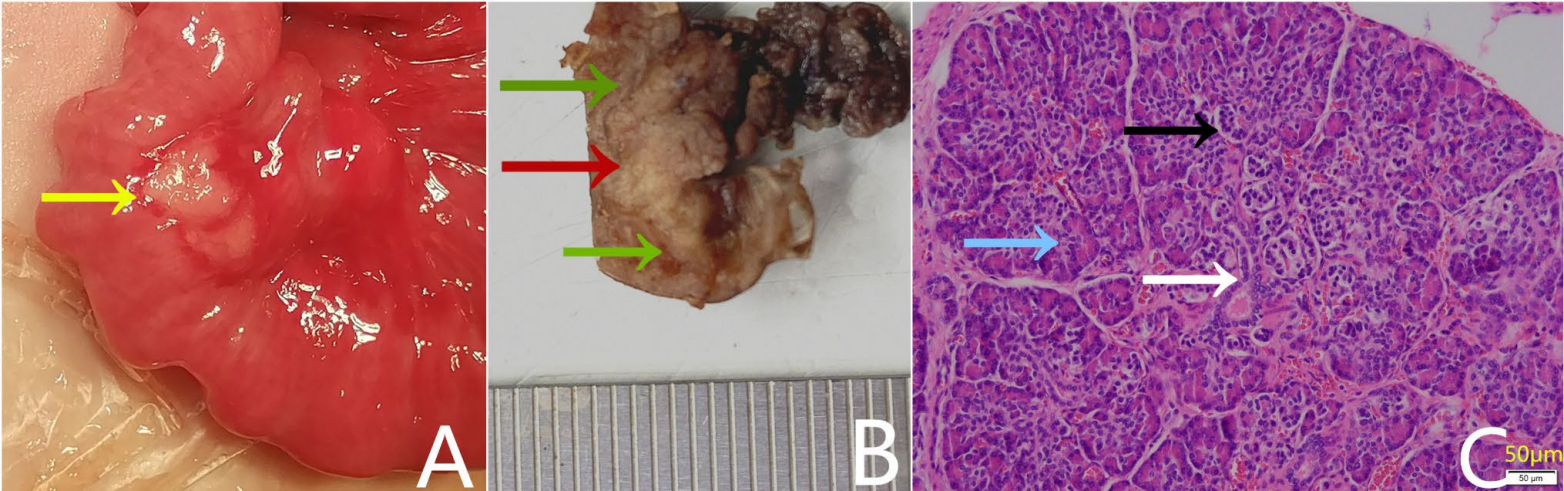
A 2-day-old boy with malrotation complicating midgut volvulus. (**A**) intraoperative findings showing a mass at the jejunal mesentery (yellow arrow) (**B**) gross pathologic appearance revealing an irregular mass along the mesenteric border (red arrow) compressing the adjacent jejunal wall (green arrows) (**C**) pathologic findings confirming heterotopic pancreas (Heinrich type I), including pancreatic acini (blue arrow), duct (white arrow), and islet cells (black arrow). (Hematoxylin–eosin stain, original magnification, 200 ×)

**Fig. 2:**
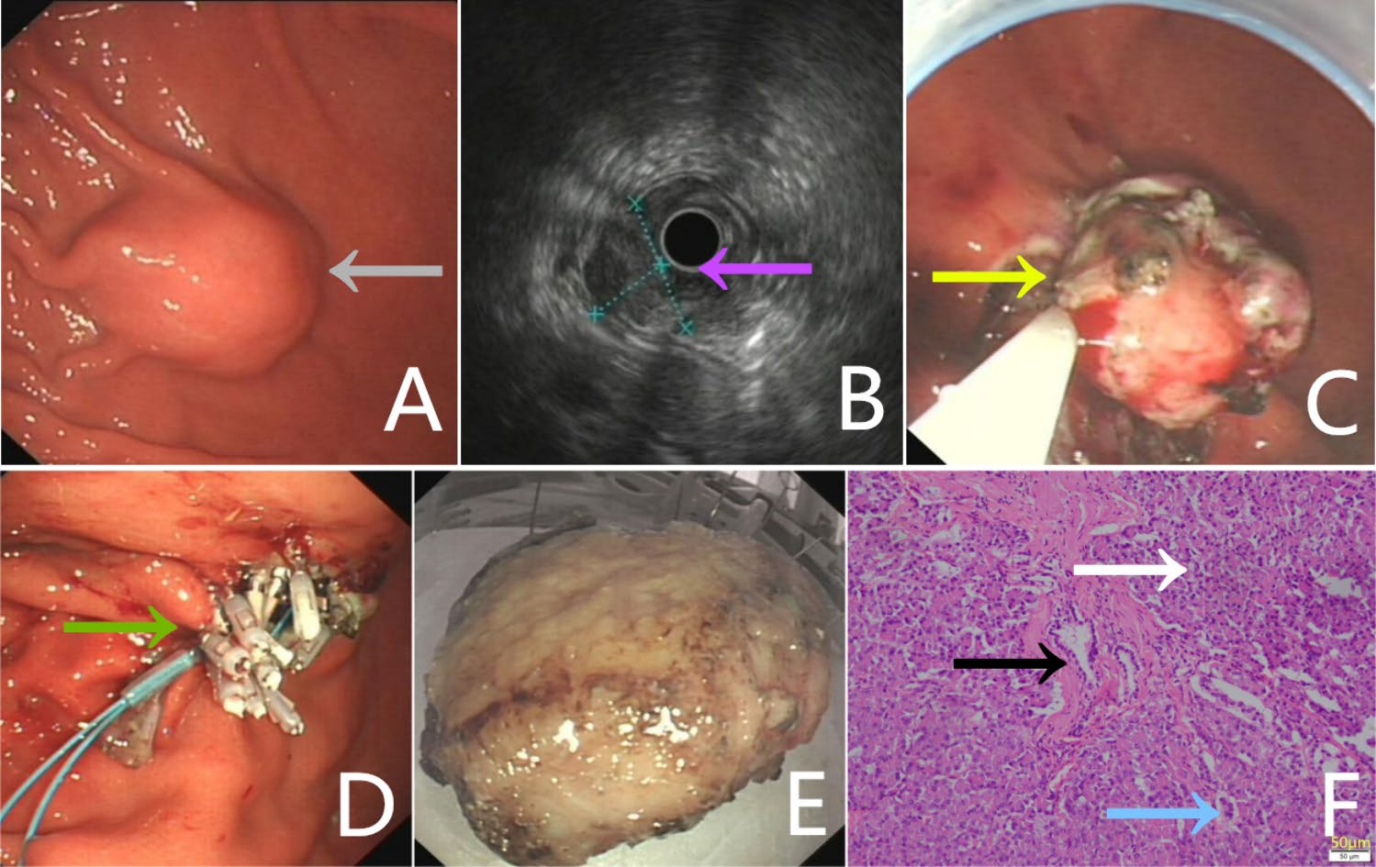
An 18-year-old boy with recurrent epigastric pain and non-bilious vomiting (**A**) gastroscopic view of a polypoid submucosal mass (gray arrow) with intact overlying mucosa located in the greater curvature of the stomach fundus (**B**) endoscopic ultrasonography (EUS) showing a submucosal mass heterogeneous and hypoechoic (pink arrow), 2.5 cm × 2.0 cm in size, being suspicious for stromal tumor (**C**) ESD procedure, dissecting the mucosa to expose the submucosal mass (yellow arrow), removing the slight yellow-colored multilobulated massclip closure (green arrow) (**E**) ESD resected sample(**F**) pathological findings confirming heterotopic pancreas (Heinrich type I), including pancreatic acini (white arrow), duct (black arrow), and islet cells (blue arrow), (hematoxylin–eosin, original magnification, 200 ×)

**Fig. 3:**
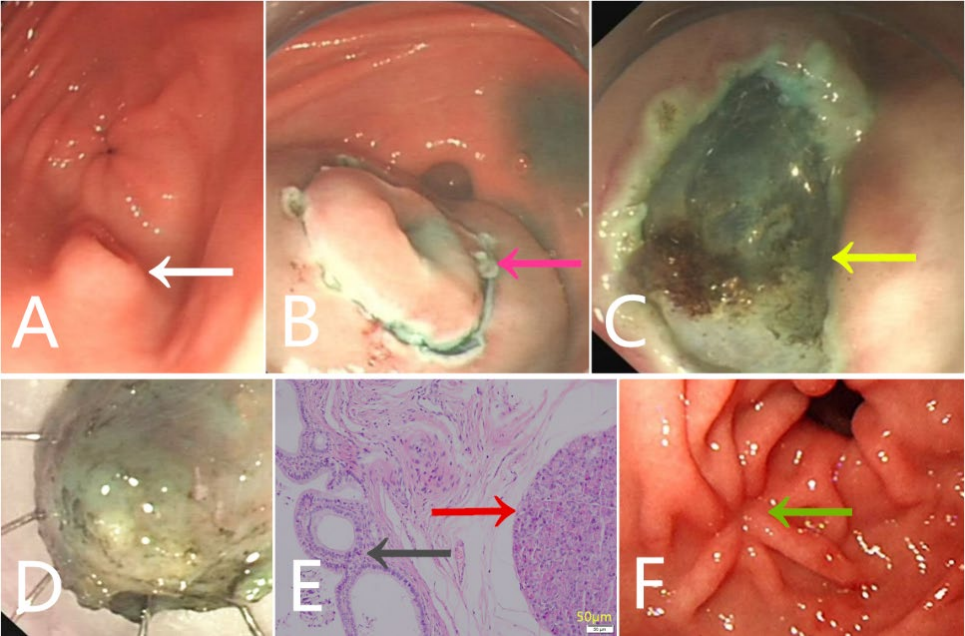
A 13-year-old boy with chronic epigastric pain. (**A**) an antral umbilicated submucosal nodule (white arrow) protruding into the gastric lumen under gastroscopy, suggestive of submucosal heterotopic pancreas (**B**) ESD technique (pink arrow) (**C**) defect after removal of the nodule (yellow arrow)clip closure post-ESD referring to Fig. 1D (**D**) ESD resected sample (**E**) pathological findings confirming heterotopic pancreas (Heinrich type II), including pancreatic acini (red arrow) and ducts (gray arrow). (Hematoxylin–eosin stain, original magnification, 200 ×). (**F**) appearance of endoscopic follow up 11 weeks after ESD (green arrow)

Management approaches included open surgery (Fig. 1), laparoscopic or laparoscopic-assisted mini-incision surgery for intra-abdominal lesions, endoscopic submucosal dissection (ESD) for gastric heterotopic pancreas (Figs. 2 and 3), and surgical removal of the umbilical lesions. Curative treatments included segmental bowel resection (*n* = 49), gastrointestinal wedge resection (*n* = 12), mass resection (*n* = 20), ESD (*n* = 6), and endoscopic biopsy (*n* = 1).

No postoperative hemorrhage, ESD perforations or bleeding, surgical site infection or organ/space infection, incision dehiscence, bowel anastomotic leak or stenosis, and adhesive small bowel obstruction related to the management of heterotopic pancreas occurred over six months to one year follow-up period.

## Discussion

Heterotopic pancreas is defined as pancreatic tissue existing in an abnormal location and lacking any direct or vascular communication with the normal pancreas [6, 13, 14]. Its pathogenesis remains unclear.7 Based on the pathological classification, heterotopic pancreas is divided into three pathological types [11]. Type I consists of three components: acini, duct, and islet cellstype II is composed of acini and fewer ductsand type III consists of only pancreatic ducts. Some studies have revealed that gastrointestinal adenomyoma is a variant of type III heterotopic pancreas evidenced by the epithelial component resembling the pancreatic duct and the absence of acini formation [15]. For instance, Rhim et al. [16]. reported an 8-week-old infant who presented with obstruction due to gastric adenomyoma. In our study, two cases of adenomyoma were identified, including one concomitant with type I heterotopic pancreas. However, others consider adenomyomas to be myoepithelial hamartomas, an independent disorder with nonspecific symptoms [17].

The prevalence of heterotopic pancreas ranges from 0.6 to 13%, and with the widespread use of endoscopy screening, heterotopic pancreas is not a rare finding [5, 7, 13]. Clinical data analysis from our case series revealed that there was a slight predominance of males (1.84:1), with nearly half (46.59%) of the patients aged 1 year or younger.

Our case series confirmed previous findings that the heterotopic pancreas is commonly located at the apex of Meckel’s diverticulum, jejunum, umbilicus, ileum, and stomach [12, 18–22]. Heterotopic pancreatic tissues found within the Meckel’s diverticulum often accompany gastric mucosa heterotopia (80.49%), a finding seldom reported in the literature, with its pathogenesis remaining unclear [23, 24].

Regarding symptomatology, nearly 75% of the cases with heterotopic pancreas were incidentally diagnosed during other abdominal surgical procedures, consistent with previous reports [25]. The prevalence of symptoms related to heterotopic pancreas in patients aged 5 years and older was 53.33% compared to those under 5 years of age (19.18%). These results are consistent with the findings of Persano et al. [5]. Common symptoms included mechanical obstruction, bleeding, and umbilical discharge in the pediatric population [21]. Notably, heterotopic pancreas in the stomach may lead to clinical symptoms more often than in other sites. However, no evidence of neoplasia was observed in this study, although malignant transformation may complicate heterotopic pancreas in adults and adolescents [1, 5, 7].

The preoperative diagnosis of symptomatic heterotopic pancreas is challenging, and it should be considered in the differential diagnosis of acute abdominal pain, inflammation, obscure gastrointestinal bleeding or perforation, bowel or biliary obstruction, and malignant tumors [1, 19, 25]. Ginsburg et al. [26]. described mesenteric ectopic pancreatitis in an adolescent with acute abdominal pain and elevated serum lipase and amylase levels. Massive gastrointestinal bleeding caused by heterotopic pancreas is rare and is usually confirmed by endoscopy, CT enterography, laparoscopic exploration, exploratory laparotomy, intraoperative endoscopy, and even capsule endoscopy [20]. In our case series, twenty-five Meckel’s diverticulum cases presented with lower gastrointestinal bleeding, and only three were defined as heterotopic pancreas-associated bleeding. Heterotopic pancreas should be considered in the differential diagnosis among patients with lower gastrointestinal bleeding after ruling out Meckel’s diverticulum with GMH [20]. It should also be considered in children with umbilical discharge [19]. Ultrasonography and fistulogram may help in making an accurate diagnosis.

Heterotopic pancreas may present with a variety of symptoms requiring surgical intervention [4, 5, 21]. Gastric heterotopic pancreas can be misdiagnosed as other submucosal masses, such as stromal tumors. Imaging techniques like contrast CT and MRI can assist in making accurate diagnoses while EUS can show the origination and hypoechoic or mixed pattern of the heterotopic pancreas in the layers of the gastrointestinal wall [27–29].

Regarding management options, surgical removal of the heterotopic pancreas should be considered due to its potential complications, including inflammation, ulceration, gastrointestinal bleeding or perforation, bowel or biliary obstruction, and even malignant transformation with increasing age [8, 26]. In our present study, foci of the heterotopic pancreas were removed in all cases with ESD, laparoscopic, laparoscopic-assisted, or open surgical approach based on patients’ age, the lesion site and size, and co-existing diseases. Although over 86% of cases were treated with open surgery in this case series, minimal invasive approach should be considered as a treatment option in selected cases in the era of minimal access surgery [18, 29]. What’s more, EUS examination combined with extended biopsy or endoscopic SPOT^®^ Tattooing may help decide the type of procedure in submucosal gastric heterotopic pancreas [30–33]. In recent years, the characteristic imaging features and precise preoperative diagnosis may help differentiate heterotopic pancreas from malignancies, thus avoiding unnecessary extensive surgical intervention [6].

Regarding the occurrence of intra-operative complications for excision of heterotopic pancreas, surgeons may face intra-operative technical challenges, such as performing complex and multiple procedures, intracorporeal suturing skills, conversion to open or laparoscopic repair, prolonged operative time and anesthesia time [34–36]. In our case series, one neonatal case had malrotation and midgut volvulus with incidental large mesenteric heterotopic pancreas, which was really decision-making dilemmas for surgeons. Excision of heterotopic pancreas with involved bowel segment and primary anastomosis was performed. Although the patient recovered uneventfully, longer operative time and anesthesia time, multiple procedures, and requirements of high experienced skills may increase intra-operative risks and postoperative complications in the newborn period. To ensure these patients in a stable condition and avoid potential intra- and postoperative complications, staged procedures [34] should be considered in both hemodynamically unstable and stable patients.

Regarding the postoperative complications of heterotopic pancreas, like other surgical procedures, infection (superficial incisional, deep incisional, and organ/space), incision dehiscence, bleeding, anastomotic leak/stenosis, or adhesive small bowel obstruction may occur in patients with heterotopic pancreas located in intestine or in mesentery involving bowel segment [36–39].

Our study has limitations, including its retrospective study design, somewhat small number of patients, and heterogeneity due to data collection from four different institutions over a longer period of time. A more diverse pool of cases may obtain a more representative sample and improve the generalizability of our experience.

## Conclusions

Our multicenter retrospective study revealed that only 25% of the patients presented with symptoms. However, 75% of the cases had comorbidities, with the majority of the cases of Meckel’s diverticulum being accompanied by GMH. Surgical removal of the lesion is recommended due to its potential complications, highlighting the need for prospective multi-center studies to establish more rational treatment options, particularly in asymptomatic small babies or patients with severe coexisting diseases.

## Data Availability

Anonymus data can be provided upon request to the corresponding author.
